# Mutual enrichment in ranked lists and the statistical assessment of position weight matrix motifs

**DOI:** 10.1186/1748-7188-9-11

**Published:** 2014-04-05

**Authors:** Limor Leibovich, Zohar Yakhini

**Affiliations:** 1 Department of Computer Science, Technion - Israel Institute of Technology, Technion City, Haifa 32000, Israel; 2 Agilent Laboratories Israel, 94 Em Hamoshavot Road, 49527 Petach-Tikva, Israel

**Keywords:** Statistical enrichment, Position weight matrices, High-throughput sequencing data analysis, Tissue specific methylation patterns, lncRNA

## Abstract

**Background:**

Statistics in ranked lists is useful in analysing molecular biology measurement data, such as differential expression, resulting in ranked lists of genes, or ChIP-Seq, which yields ranked lists of genomic sequences. State of the art methods study fixed motifs in ranked lists of sequences. More flexible models such as position weight matrix (PWM) motifs are more challenging in this context, partially because it is not clear how to avoid the use of arbitrary thresholds.

**Results:**

To assess the enrichment of a PWM motif in a ranked list we use a second ranking on the same set of elements induced by the PWM. Possible orders of one ranked list relative to another can be modelled as permutations. Due to sample space complexity, it is difficult to accurately characterize tail distributions in the group of permutations. In this paper we develop tight upper bounds on tail distributions of the size of the intersection of the top parts of two uniformly and independently drawn permutations. We further demonstrate advantages of this approach using our software implementation, mmHG-Finder, which is publicly available, to study PWM motifs in several datasets. In addition to validating known motifs, we found GC-rich strings to be enriched amongst the promoter sequences of long non-coding RNAs that are specifically expressed in thyroid and prostate tissue samples and observed a statistical association with tissue specific CpG hypo-methylation.

**Conclusions:**

We develop tight bounds that can be calculated in polynomial time. We demonstrate utility of mutual enrichment in motif search and assess performance for synthetic and biological datasets. We suggest that thyroid and prostate-specific long non-coding RNAs are regulated by transcription factors that bind GC-rich sequences, such as EGR1, SP1 and E2F3. We further suggest that this regulation is associated with DNA hypo-methylation.

## Background

Modern data analysis often faces the task of extracting characteristic features from sets of elements singled out according to some measurement. In molecular biology, for example, an experiment may lead to measurement results pertaining to genes and then questions are asked about the properties of genes for which these were high or low. This is an example, of course, and the set of elements does not have to be genes. They can be genomic regions, proteins, structures, etc. A central technique for addressing the analysis of characteristic properties of sets of elements is statistical enrichment. More specifically – the experiment results are often representable as ranked lists of elements and we then seek enrichment of other properties of these elements at the top or bottom of the ranked list. GSEA [1], for example, is a tool that addresses characteristic features of genes that are found to be differentially expressed in a comparative transcriptomics study. GOrilla [2,3] addresses GO terms enriched in ranked lists of genes where the ranking can be, for example, the result of processing differential expression data or of correlations computed between genomic DNA copy number and expression [4-6]. FATIGO [7] is also a tool that is useful in the context of analysing GO terms in ranked lists of genes. DRIMust [8-10] searches for sequence motifs that are enriched, in a statistically significant manner, in the top of a ranked list of sequences, which can be produced by techniques like ChIP-Seq.

All the aforementioned tools utilize a statistical approach that is based on assessing enrichment of an input set in an input ranked list by quantifying the enrichment obtained at various cutoffs applied to the ranked list. It is often the case, however, that two quantitative properties need to be compared to each other. For example, when the elements are genes, we may have measured differential expression values, as well as measured ChIP-Seq signals. We are therefore interested in assessing mutual enrichment in two ranked lists. Another example consists of one ranking according to differential expression and one according to prediction scores for miRNA targets. miTEA [11,12] addresses this latter case by statistically assessing the enrichment of miRNA targets in a ranked list of genes (also see [13]). To address mutual enrichment in two ranked lists over the same set of *N* elements, miTEA [11] performs analysis on permutations. Mutual enrichment in the top of two ranked lists can be simplified, from a mathematical point of view, by arbitrarily setting the indices of one list to the identity permutation (1,2,…,*N*) and treating the other list as a permutation *π* = *π*(1), …, *π*(*N*) over these numbers. For the purpose of assessing the intersection of the top of the two ranked lists in a data driven manner, miTEA asks which prefix [1,…,*n*_1_] is enriched in the first *n*_2_ elements of *π*, that is in the set *π*(1), …, *π*(*n*_2_). The statistics introduced by miTEA is called mmHG (minimum-minimum-Hyper-Geometric). A slightly different variant of mmHG is described later in this section.

Statistics in the group of permutations S_*N*_ is often difficult because the number of entities to be considered by any null model is *N*!. Direct exhaustive calculation of tail distributions over S_*N*_ is therefore impractical for all but very small values of *N*. This difficulty is addressed by several heuristic techniques. Mapping into continuous spaces, such as in [14], has proven useful in certain cases but not for studying large deviations. In the case of enrichment statistics that focuses on the top of the permutation and seeks to assess extreme events, such as mmHG, we prefer to use bounds on tail probabilities. Tail probabilities are useful constructs when applied to analysing molecular biology measurement data as they enable statistical assessment of observed results.

In this work we derive tight bounds on the tail probabilities of mutual enrichment at the top of two random permutations uniformly drawn over S_*N*_ and demonstrate the utility of this approach in the context of flexible motif discovery. Our bounds are computable in polynomial time and potentially add to the accuracy of reported position weight matrix (PWM) motifs for nucleic acid sequences.

### Mutual enrichment in ranked lists – the mmHG statistics

The mmHG statistics [11] is a generalization of the mHG statistics [2,15-17]. The mHG statistics quantifies the enrichment level of a set of elements at the top of a ranked list of elements of the same type, whereas the mmHG statistics assesses the level of mutual enrichment in two ranked lists over the same set of elements. While any parametric or non-parametric correlation statistics (e.g. Spearman’s correlation coefficient), that takes the same input, calculates the overall agreement between the two ranked lists, the mmHG statistic focuses only on agreement at the top of the two ranked lists. mmHG counts elements common to the top of both lists, without predefining what top is. Its intended output is the probability of observing an intersection at least as large in two randomly ranked lists (defined as the enrichment *p*-value). In this section we describe the mmHG statistics and in later sections we suggest tight bounds for the *p*-value. Our definition of the mmHG statistics varies slightly from that of Steinfeld *et al.*[11], which is used by miTEA.

Mutual enrichment in the top of two ranked lists can be simplified, from a mathematical point of view, by arbitrarily setting the indices of one list to the identity permutation (1,2,…,*N*) and treating the other list as a permutation. Details of this transform are given in the next section. We now define mmHG for the simple case of one permutation. Consider a permutation *π* = *π*(1), …, *π*(*N*) ∈ *S*_*N*_ - the group of all permutations over the numbers 1,…,*N*. mmHG is a function that takes *π* and calculates two numbers 1 ≤ *n*_1_, *n*_2_ ≤ *N* such that the observed intersection between the numbers 1,…,*n*_1_ and the first *n*_2_ elements of *π* – namely, *π*(1), …, *π*(*n*_2_) - is the most surprising in terms of the hypergeometric *p*-value.

Formally, given *π* ∈ *S*_*N*_ and for every 1 ≤ *n*_1_, *n*_2_ ≤ *N*, let *b*_*π*_(*n*_1_, *n*_2_) be the size of the intersection of 1,…,*n*_1_ with *π*(1), …, *π*(*n*_2_). Set

$$\begin{array}{l} \mathrm{mmHG}\;\mathrm{score}\left(\pi \right)\\ \;=\mathrm{mi}n_{1\leq n_{1}\leq N}\mathrm{mi}n_{1\leq n_{2}\leq N}\mathrm{HGT}\left(N,n_{1},n_{2},b_{\pi }\left(n_{1},n_{2}\right)\right) \end{array}$$

where HGT is the tail distribution of an hypergeometric random variable:

$$\mathrm{HGT}\left(N,n_{1},n_{2},b\right)=\sum_{i=b}^{\min\left(n_{1},n_{2}\right)}\frac{\left(\begin{array}{c} n_{1}\\ i \end{array}\right)\left(\begin{array}{c} N-n_{1}\\ n_{2}-i \end{array}\right)}{\left(\begin{array}{c} N\\ n_{2} \end{array}\right)}$$

The mmHG score cannot be considered as a significance measure, due to the multiple testing involved in finding *n*_1_ and *n*_2_. A simple way to correct an mmHG score *s* for multiple testing and report an upper bound on the *p*-value is to use the Bonferroni correction. Basically, *s* is multiplied by the number of multiple tests conducted (which is *N*^2^), yielding an upper bound on the p-value, as follows:

$$\mathrm{mmHG}\;p-\mathrm{value}\left(s,N\right)\leq s\cdot N^{2}$$

In the Results section we present significantly tighter bounds.

### Position weight matrix motifs

Data produced by techniques such as ChIP-Seq [18], ChIP-exo [19], CLIP [20], PAR-CLIP [21] and others are readily representable as ranked lists of sequences, where the ranking is according to the measured binding affinity. Computational tools and approaches to motif discovery form part of the data analysis workflow that is used to extract knowledge and understanding from this type of studies. We are often interested in sequence motifs that are observed to be enriched in sequences where strong binding affinity is measured. A position weight matrix (PWM) is a commonly used representation of motifs in biological sequences [22-24]. This representation is more faithful to the underlying biology than representation by exact words, owing to the tendency of binding sites to be short and degenerate [25]. A PWM is a matrix of score values that gives a weighted match to any given substring of fixed length. It has one row for each symbol in the alphabet, and one column for each position in the pattern. Assuming an input sequence of length equal to the PWM width, we simply multiply the scores assigned to each letter in each of the positions in the input sequence to obtain the likelihood of the input string (alternatively, we can sum the logarithms of the probabilities). That is, the score assigned by a PWM to a substring *S* = *S*_1_…*S*_K_ is defined as $\prod_{j=1}^{K}p_{s_{j},j}$, where *j* represents a position in the substring; *s*_*j*_ is the symbol at position *j* in the substring; and *p*_*α*,*j*_ is the score in row *α*, column *j* of the matrix. In other words, a PWM score is the product of position-specific scores for each symbol in the substring. This definition can be generalized to yield a score for a sequence *S* = *S*_1_…*S*_M_ longer than the PWM by calculating $\mathrm{ma}x_{1\leq i\leq M-K+1}\prod_{j=1}^{K}p_{s_{i+j-1},j}$. Alternatively, an enhanced model that takes into account multiple occurrences of the PWM in the sequence can be applied by summing over sufficiently strong occurrences of the PWM or by other more sophisticated approaches [26].

### mmHG statistics for PWM motifs

Given a set of sequences that were tested in a high throughput experiment such as ChIP-Seq [18], CLIP [20] and others, they can be ranked according to the measured binding affinities, yielding a ranked list *L*_1_. Since usually we are interested in finding motifs amongst sequences having strong binding affinities, we actually search for motifs that are more prevalent at the top of this list. It is clear that any algorithm for de-novo flexible motif search would need to evaluate candidate PWMs. Given a PWM which we want to assess, the sequences can also be ranked according to their PWM scores, yielding another ranked list *L*_2_, different from *L*_1_. A significant PWM motif would yield significant scores for sequences having strong binding affinities. Therefore, the question of PWM motif discovery from ranked experimental data can be formulated as quantifying the mutual enrichment level for the two ranked lists *L*_1_ and *L*_2_. Given two ranked lists *L*_1_ and *L*_2_ over the universe of *N* sequences, they can be transformed into two respective permutations, *π*_1_ = (*π*_1_(1), …, *π*_1_(*N*)) and *π*_2_ = (*π*_2_(1), …, *π*_2_(*N*)). The relative permutation *π*, of *π*_2_ w.r.t. *π*_1_, is defined by *π*(*π*_1_(*j*)) = *π*_2_(*j*), for every *j* = 1,…,*N*, or simply, using operations in the group S_*N*_: *π* = *π*_2_ ∙ *π*_1_^- 1^. Using the relative permutation *π*, we can represent the mutual enrichment of the top parts of *L*_1_ and *L*_2_ as *mmHG score* (*π*), defined above.

## Results

### Estimation of the mmHG *p*-value – introducing first upper bound – B1

Given an mmHG score *s*, observed in analysing real measurement data, we would like to assess the statistical significance of this observation. Assuming endless computational power, we would enumerate all permutations and calculate the mmHG score for each, in order to characterize the distribution of mmHG as a random variable over S_*N*_. The *p*-value for *s* is then simply:

$$\begin{array}{l} \mathrm{mmHG}\;p-\mathrm{value}\left(s,N\right)\\ \;=\frac{\mathrm{The}\;\mathrm{number}\;\mathrm{of}\;\mathrm{permutations}\;\mathrm{having}\;\mathrm{mmHG}\;\mathrm{score}\leq s}{N!} \end{array}$$

Since the number of permutations is huge, the process described above is very far from feasible. Therefore, we seek a computationally tractable upper bound, preferably tight.

A trivial upper bound is the Bonferroni corrected mmHG score defined by *s* · *N*^2^. A more subtle upper bound was suggested by Steinfeld *et al.*[11] and is briefly described later as bound B3. In this work we introduce tighter bounds that are polynomially computable.

We next describe an intuitive upper bound (B1) which we later refine to produce a tighter bound (B2). The input of the problem consists of an mmHG score *s* and the total number of elements *N*. The output is an upper bound on the *p*-value. The efficiency of our approach relies on enumerating all possible HGT scores rather than enumerating all permutations in S_*N*_. This approach is computationally efficient as HGT is a function of four input parameters: *N*, *n*_*1*_, *n*_*2*_, and *b*. Given *N*, there are O(*N*^3^) possible combinations of *n*_*1*_, *n*_*2*_, and *b*. Also, given *N*, *n*_1_ and *n*_2_, *b* can be any integer in the range [max(0, *n*_2_ - *N* + *n*_1_),   min(*n*_1_, *n*_2_)]. Next, all is left to do is to determine how many permutations correspond to each HGT score. To this end, let Λ(*N, n*_1_*, n*_2_*, b*) be the number of permutations for which it holds that *b* out of the first *n*_2_ entries in the permutation are taken from the range [1,…,*n*_1_]. This formulation is equivalent to counting permutations for which we attain, at some point, the value HGT(*N*, *n*_1_, *n*_2_, *b*), had we taken the exhaustive approach. Λ(*N, n*_1_*, n*_2_*, b*) can be represented as:

$$\Lambda \left(N,n_{1},n_{2},b\right)=\left(\begin{array}{c} n_{1}\\ b \end{array}\right)\left(\begin{array}{c} n_{2}\\ b \end{array}\right)b!\left(\begin{array}{c} N-n_{1}\\ n_{2}-b \end{array}\right)\left(n_{2}-b\right)!\left(N-n_{2}\right)!$$

as we first choose *b* elements from the range [1,…,*n*_1_] to appear at the first *n*_2_ entries of the permutation (there are $\left(\begin{array}{c} n_{1}\\ b \end{array}\right)$ possibilities). Then, we choose the positions amongst the first *n*_2_ entries that are occupied by these *b* elements, while considering all internal arrangements (for each choice of *b* elements there are $\left(\begin{array}{c} n_{2}\\ b \end{array}\right)b!$ possibilities). We next choose *n*_2_-*b* elements from the range [*n*_1_ + 1,…,*N*] to appear at the rest of the first *n*_2_ entries of the permutation (there are $\left(\begin{array}{c} N-n_{1}\\ n_{2}-b \end{array}\right)$ possibilities for that) and consider all possible (*n*_2_ - *b*) ! arrangements. Finally, we take into account all possible (*N* - *n*_2_) ! arrangements of the remaining *N*-*n*_2_ entries of the permutation.

A straightforward upper bound for the number of permutations in S_*N*_ having mmHG score better than *s* follows:

$$|\left\{\pi^{'}\epsilon S_{N}:\mathrm{mmHG}\left(\pi^{'}\right)\leq s\right\}|\leq \sum_{n_{1},n_{2},b:\mathrm{HGT}\left(N,n_{1},n_{2},b\right)\leq s}\Lambda \left(N,n_{1},n_{2},b\right)$$

From which an upper bound is easily derived:

$$\mathrm{mmHG}\;p-\mathrm{value}\left(s,N\right)\leq \frac{\sum_{n_{1},n_{2},b:\mathrm{HGT}\left(N,n_{1},n_{2},b\right)\leq s}\Lambda \left(N,n_{1},n_{2},b\right)}{N!}$$

By algebraic manipulations we get:

$$\mathrm{mmHG}\;p-\mathrm{value}\left(s,N\right)\leq \sum_{n_{1},n_{2},b:\mathrm{HGT}\left(N,n_{1},n_{2},b\right)\leq s}\frac{\left(\begin{array}{c} n_{1}\\ b \end{array}\right)\left(\begin{array}{c} N-n_{1}\\ n_{2}-b \end{array}\right)}{\left(\begin{array}{c} N\\ n_{2} \end{array}\right)}$$

This upper bound is simple and requires O(*N*^3^) HGT calculations. An HGT calculation takes O(*N*) time, assuming binomial coefficients can be calculated in constant time. Constant time computation can be achieved using Stirling’s approximation [27]: $\sqrt{2\mathrm{\pi n}}{\left(\frac{n}{e}\right)}^{n}e^{\frac{1}{12n+1}}\leq n!\leq \sqrt{2\mathrm{\pi n}}{\left(\frac{n}{e}\right)}^{n}e^{\frac{1}{12n}}$, which is tight for large factorials.

### A refined upper bound for the *p*-value – B2

The upper bound introduced in the previous section counts the number of permutations for which the value HGT(*N*, *n*_1_, *n*_2_, *b*) is calculated when taking the non-practical exhaustive approach that enumerates over all *N*! permutations. Ideally, we wish to count the number of permutations for which the value HGT(*N*, *n*_1_, *n*_2_, *b*) is also their mmHG score, as a permutation may correspond with many HGT values that are better than *s*, so it can be counted more than once. This explains why the formula introduced earlier is an upper bound and not an exact *p*-value. A second observation that follows is that the smaller the mmHG score *s*, the tighter the bound, because a permutation will have fewer combinations (*N*, *n*_1_, *n*_2_, *b*) having HGT values better than *s*.

Therefore, if we can reduce the extent of multiple counting of the same permutation, we will get a tighter bound. We do this by looking one step backwards. If, for example, HGT(*N*, *n*_1_, *n*_2_, *b*) ≤ *s*, we can exclude from the counting permutations that contain *b* elements from the range [1,…,*n*_1_-1] at their first *n*_2_ entries because they are already taken into account in Λ(*N*, *n*_1_ - 1, *n*_2_, *b*) (because necessarily HGT(*N*, *n*_1_ - 1, *n*_2_, *b*) ≤ *s*, as we will later explain).

Let ψ(*N*, *n*_1_, *n*_2_, *b*) be the set of permutations for which it holds that *b* out of the first *n*_2_ entries are taken from the range [1,…,*n*_1_] (note that Λ(*N*, *n*_1_, *n*_2_, *b*) introduced earlier is, in fact, the size of ψ(*N*, *n*_1_, *n*_2_, *b*)). Assuming HGT(*N*, *n*_1_, *n*_2_, *b*) ≤ *s*, we can partition the set ψ(*N*, *n*_1_, *n*_2_, *b*) into five disjoint subsets *ψ*_1_, …, *ψ*_5_ such that *ψ* = *ψ*_1_ ∪ *ψ*_2_ ∪ *ψ*_3_ ∪ *ψ*_4_ ∪ *ψ*_5_, as follows:

$$\begin{array}{ll} \;\psi_{1}=\psi \left(N,n_{1},n_{2},b\right) & \cap \psi \left(N,n_{1}-1,n_{2}-1,b-1\right)\cap \psi \left(N,n_{1}-1,n_{2},b\right)\\ \;\psi_{2}=\psi \left(N,n_{1},n_{2},b\right) & \cap \psi \left(N,n_{1}-1,n_{2}-1,b-1\right)\cap \psi \left(N,n_{1},n_{2}-1,b\right)\\ \;\psi_{3}=\psi \left(N,n_{1},n_{2},b\right) & \cap \psi \left(N,n_{1}-1,n_{2}-1,b-1\right)\cap \psi \left(N,n_{1}-1,n_{2},b-1\right)\\ & \cap \psi \left(N,n_{1},n_{2}-1,b-1\right)\\ \;\psi_{4}=\psi \left(N,n_{1},n_{2},b\right) & \cap \psi \left(N,n_{1}-1,n_{2}-1,b\right)\\ \;\psi_{5}=\psi \left(N,n_{1},n_{2},b\right) & \cap \psi \left(N,n_{1}-1,n_{2}-1,b-2\right)\\ & \cap \psi \left(N,n_{1}-1,n_{2},b-1\right)\cap \psi \left(N,n_{1},n_{2}-1,b-1\right) \end{array}$$

The properties of the hypergeometric distribution imply that given a tuple (*N*, *n*_1_, *n*_2_, *b*), the permutations in *ψ*_1_, *ψ*_2_, *ψ*_4_ can be disregarded from the current counting iteration. To explain why, we will demonstrate the argument on *ψ*_1_. The permutations in *ψ*_1_ contain *b* elements from the range [1,…,*n*_1_-1] at their first *n*_2_ entries. Recall that we also assume that HGT(*N*, *n*_1_, *n*_2_, *b*) ≤ *s*. Therefore HGT(*N*, *n*_1_ - 1, *n*_2_, *b*) ≤ *s* also holds, as the same intersection is observed for even a smaller set. Thus, the permutations in *ψ*_1_ have already been counted as having HGT value better than *s* when handling the triplet *n*_1_-1, *n*_2_ and *b*, and can be disregarded for the combination of *n*_1_, *n*_2_ and *b*. Similar arguments hold for *ψ*_2_ and *ψ*_4_.

The permutations in *ψ*_3_ should be counted if three conditions hold: the first is HGT(*N*, *n*_1_ - 1, *n*_2_ - 1, *b* - 1) > *s*; the second is HGT(*N*, *n*_1_ - 1, *n*_2_, *b* - 1) > *s*; and the third is HGT(*N*, *n*_1_, *n*_2_ - 1, *b* - 1) > *s*. Otherwise, the permutations in *ψ*_3_ have been counted by former triplets. Similarly, the permutations in *ψ*_5_ should be counted if the following three conditions hold: HGT(*N*, *n*_1_ - 1, *n*_2_ - 1, *b* - 2) > *s*, HGT(*N*, *n*_1_ - 1, *n*_2_, *b* - 1) > *s*, and HGT(*N*, *n*_1_, *n*_2_ - 1, *b* - 1) > *s*. Finally, we calculate the sizes of *ψ*_3_ and *ψ*_5_, in the relevant cases. The definition of *ψ*_3_ implies that it consists of permutations that contain *b*-1 elements taken from the range [1,…,*n*_1_-1] at their first *n*_2_-1 entries, and also *n*_1_ is positioned at entry *n*_2_. Therefore:

$$\left|\psi_{3}\right|=\left(\begin{array}{c} n_{1}-1\\ b-1 \end{array}\right)\left(\begin{array}{c} n_{2}-1\\ b-1 \end{array}\right)\left(b-1\right)!\left(\begin{array}{c} N-n_{1}\\ n_{2}-b \end{array}\right)\left(n_{2}-b\right)!\left(N-n_{2}\right)!$$

Equivalently, the permutations in *ψ*_5_ contain *b*-2 elements taken from the subset [1,…,*n*_1_-1] at their first *n*_2_-1 entries; *n*_1_ is positioned at one of the first *n*_2_-1 entries; and entry *n*_2_ contains an element from [1,…,*n*_1_-1]. Therefore:

$$\begin{array}{ll} \;\left|\psi_{5}\right|= & \left(\begin{array}{c} n_{1}-1\\ b-2 \end{array}\right)\left(\begin{array}{c} n_{2}-1\\ b-2 \end{array}\right)\left(b-2\right)!\left(n_{2}-b+1\right)\left(\begin{array}{c} N-n_{1}\\ n_{2}-b \end{array}\right)\\ & \times \left(n_{2}-b\right)!\left(n_{1}-b+1\right)\left(N-n_{2}\right)! \end{array}$$

From the above we next conclude an upper bound. Denote

$$I\left(\mathrm{HGT}\left(N,n_{1},n_{2},b\right)>s\right)=\left\{\begin{array}{c} 1,\\ 0, \end{array}\begin{array}{c} \;\mathrm{if}\;\mathrm{HGT}\left(N,n_{1},n_{2},b\right)>s\;\\ \;\mathrm{otherwise} \end{array}\right)$$

And let Λ*(*N*, *n*_1_, *n*_2_, *b*) =

$$\begin{array}{c} \left|\psi_{3}\right|\times I\left(\mathrm{HGT}\left(N,n_{1}-1,n_{2}-1,b-1\right)>s\right)\\ \times I\left(\mathrm{HGT}\left(N,n_{1}-1,n_{2},b-1\right)>s\right)\\ \times I\left(\mathrm{HGT}\left(N,n_{1},n_{2}-1,b-1\right)>s\right)\\ +\\ \left|\psi_{5}\right|\times I\left(\mathrm{HGT}\left(N,n_{1}-1,n_{2}-1,b-2\right)>s\right)\\ \times I\left(\mathrm{HGT}\left(N,n_{1}-1,n_{2},b-1\right)>s\right)\\ \times I\left(\mathrm{HGT}\left(N,n_{1},n_{2}-1,b-1\right)>s\right) \end{array}$$

We can thus derive the following upper bound for the *p*-value:

$$\mathrm{mmHG}\;p-\mathrm{value}\left(s,N\right)\leq \frac{\sum_{n_{1},n_{2},b:\mathrm{HGT}\left(N,n_{1},n_{2},b\right)\leq s}\Lambda^{*}\left(N,n_{1},n_{2},b\right)}{N!}$$

Since Λ* is recursive, we need to define a base case. Recall that given *N*, *n*_1_ and *n*_2_, *b* can be any integer in the range [max(0, *n*_2_ - *N* + *n*_1_),   min(*n*_1_, *n*_2_)], hence determining a base case for *n*_1_ and *n*_2_ is sufficient (*N* is known). The base case here is that when *n*_1_ ≤ 1 or *n*_2_ ≤ 1, Λ*(*N*, *n*_1_, *n*_2_, *b*) is defined the same as Λ(*N*, *n*_1_, *n*_2_, *b*).

This upper bound uses more delicate counting than the bound B1 introduced in the previous section. In the following sections we assess the tightness of this bound. In later sections we demonstrate an application for PWM motif search.

### Comparison to a different mmHG variant – B3

We note that the bound described in Steinfeld *et al.*[11] addresses a slightly different variant of mmHG as a random variable over S_*N*_. The definition with which we work here is more amenable to deriving tight bounds as described above. Given a single permutation *π* ∈ *S*_*N*_ and for every *i* = 1,…,*N*, a binary vector *λ*_*i*_ is defined in which exactly *i* entries are 1 and *N*-*i* entries are 0, as follows: *λ*_*i*_(*j*) = 1 iff *π*(*j*) ≤ *i*. The mmHG score of a permutation *π* is then defined by Steinfeld *et al*. [11] as:

$$\mathrm{mmHG}\left(\pi \right)=\mathrm{mi}n_{1\leq i\leq N}P-\mathrm{value}\left(\mathrm{mHG}\left(\lambda_{i}\right)\right)\leq \mathrm{mi}n_{1\leq i\leq N}\mathrm{mHG}\left(\lambda_{i}\right)∙i$$

Where *mHG*(*λ*_*i*_) = *min*_1 ≤ *n* ≤ *N*_*HGT*(*N*, *i*, *n*, *b*_*n*_), *N* = |*λ*_*i*_| and $b_{n}=\sum_{k=1}^{n}\lambda_{i}\left(k\right)$. A possible upper bound is then given by:

$$(*)\;P-\mathrm{value}\left(\mathrm{mmHG}\left(\pi \right)\right)\leq \mathrm{mi}n_{1\leq i\leq N}\mathrm{mHG}\left(\lambda_{i}\right)\cdot i\cdot N$$

**Figure 1:**
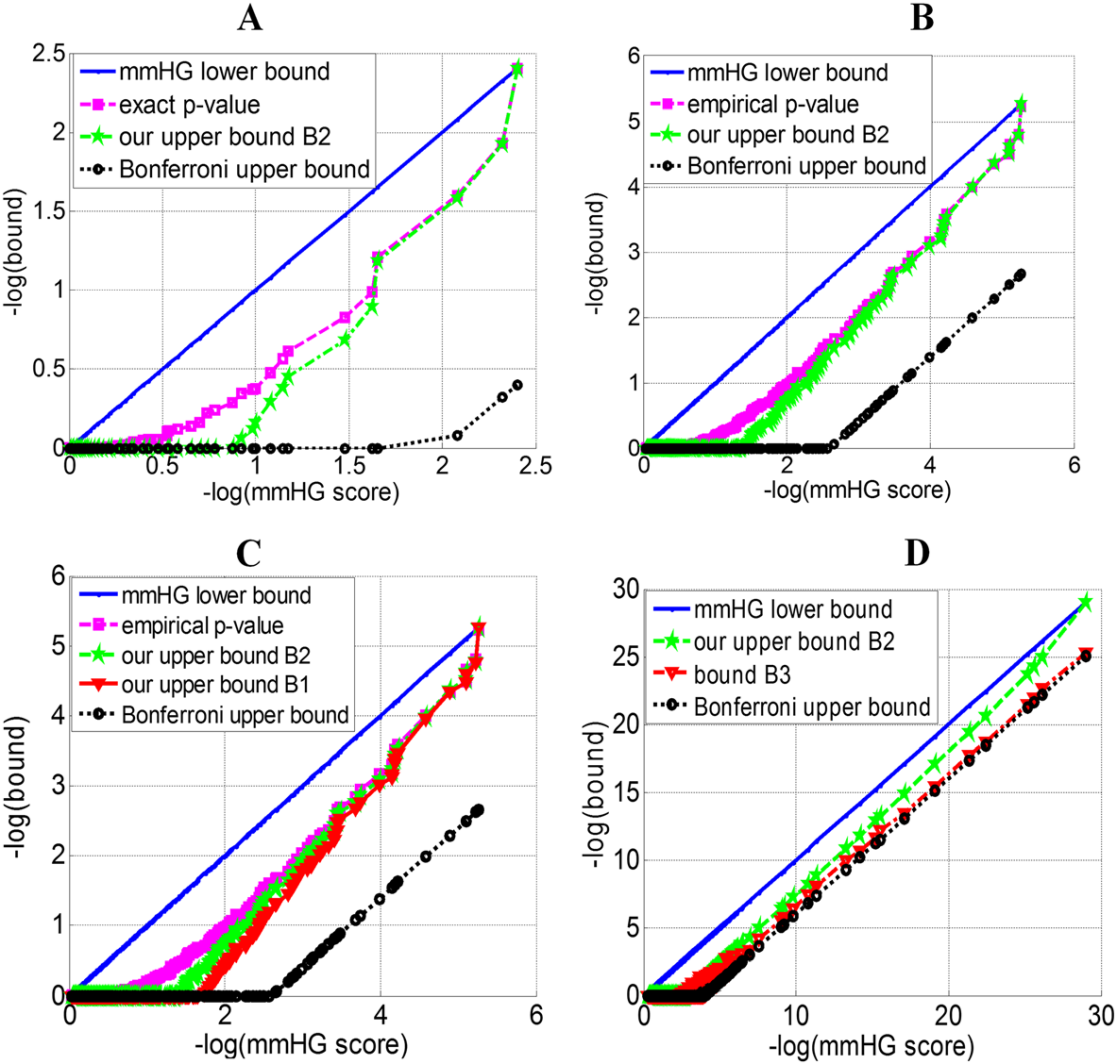
**Assessment of tightness. (A)** Four lines are shown for *N* = 10: the mmHG score, which also serves as a lower bound for the *p*-value; the exact *p*-value calculated by enumerating all 10! permutations; our refined upper bound B2; and the Bonferroni corrected *p*-value. **(B)** Here again the four lines are shown - for *N* = 20. However, instead of an exact *p*-value, which cannot be calculated exhaustively, an empirical *p*-value is produced by randomly sampling 10^7^ permutations. **(C)** In addition to the four lines shown in B, the upper bound B1 is shown (*N* = 20). **(D)** Four lines are shown for *N* = 100: the mmHG score, our upper bound B2, the bound B3 and the Bonferroni corrected *p*-value. The exact *p*-value line is positioned between the green and the blue lines. An empirical *p*-value was not calculated here as even if we sample 10^7^ permutations, a *p*-value smaller than 10^-7^ cannot be obtained.

Computing the latter quantity requires O(*N*^2^) HGT calculations, and is therefore computationally more efficient than the two bounds B1 and B2 of this current work (that require O(*N*^3^) HGT calculations). We observed that our bound B2 was tighter than the bound in (*), as later shown in Figure 1D. For example, for a permutation having mmHG score = 7.8∙10^-25^ (*N* = 100), our bound was 3.5∙10^-23^ while (*) yielded 4.2∙10^-21^. For one permutation with mmHG score = 5.1∙10^-5^ (*N* = 100), our bound was 0.026 while (*) yielded 0.2. The latter example demonstrates that a tighter bound is important for classifying an observation as statistically significant (assuming a significance threshold of 0.05).

### Assessment of tightness

In order to assess the quality of our bound B2, we compared it to the *p*-value, which can be calculated exactly for small values of *N* (that is, in cases where *N*! is not too large) and empirically for larger values of *N* (by randomly sampling permutations). Evidently, our bound B2 was significantly tighter than the Bonferroni bound for *N* = 10 (Figure 1A) and *N* = 20 (Figure 1B). We also observed that the smaller the mmHG scores – the tighter the bound, consistent with lesser over-counting for smaller scores as explained in previous sections. Furthermore, our refined bound B2 is tighter than the bound B1 (Figure 1C), and the latter is significantly better than the Bonferroni bound. Both bounds B1 and B2 are derived by enumerating HGT scores rather than enumerating permutations in S_*N*_. The refinement of this approach produced by reducing the extent of multiple counting of permutation further improves the upper bound. In addition, the bound B2 was almost always observed to be tighter than the bound B3 (Figure 1D).

### An upper bound which balances between tightness and computational cost – B4

The bound B2 is, evidently, very tight. It is, however, computationally heavy. We would still like to have an upper bound which is tighter than the Bonferroni bound and than the variant B3 but also faster to calculate. Such a compromise is achieved by generalizing an approach developed in [15] for the minimum hyper-geometric statistics. Namely, given the number of elements *N* and an attainable mmHG score *s* for which we want to calculate the *p*-value, for each 1 ≤ *b* ≤ *N* and for each 1 ≤ *n*_1_ ≤ *N*, let *n*_2_ (*b*, *n*_1_) be the maximal integer *n*_2_ so that if in a permutation *π* ∈ *S*_*N*_, *b* out of the first *n*_2_ entries in *π* are taken from the range [1,…,*n*_1_], then *π* satisfies HGT_*π*_(*N*, *n*_1_, *n*_2_, *b*) ≤ *s*. Monotonicity properties of the hyper-geometric distribution imply the existence of such *n*_2_ integers. By definition, they are constants and independent of the original permutation for which the mmHG score *s* was obtained. Due to monotonicity properties, given *b* and *n*_1_, the maximal value *n*_2_ (*b*, *n*_1_) can be calculated efficiently using binary search, which means that an upper bound that requires O(*N*^2^log*N*) calculations of HGT (instead of O(*N*^3^)) can be computed by using the following formula:

$$\mathrm{mmHG}\;p-\mathrm{value}\left(s,N\right)\leq \sum_{b,n_{1},n_{2}\left(b,n_{1}\right):\mathrm{HGT}\left(N,n_{1},n_{2}\left(b,n_{1}\right),b\right)\leq s}\frac{\left(\begin{array}{c} n_{1}\\ b \end{array}\right)\left(\begin{array}{c} N-n_{1}\\ n_{2}\left(b,n_{1}\right)-b \end{array}\right)}{\left(\begin{array}{c} N\\ n_{2}\left(b,n_{1}\right) \end{array}\right)}$$

The performance of this bound, as well as of other bounds (in terms of tightness and running time), is demonstrated in Table 1. On average, this bound was 16.5 times tighter than the Bonferroni bound; B3 was approximately 7 times tighter than Bonferroni’s bound, while B2 was 38 times tighter than Bonferroni’s, on average. The average computation time for B4 was 3 minutes, in comparison with 1 second for B3 and 26 minutes for B2. We conclude that the bound B4 presented in this section may be a good compromise between tightness and computational cost compared with the other bounds introduced in this paper.

**Table 1:** Performance of various bounds

**Protein**	**N**	**mmHG score**	**Bound B2**	**Bound B3**	**Bound B4**	**Bonferroni bound**
TNWMNG	500	2.17e-18	2.75e-14	7.5e-14	6.28e-14	5.43e-13
			0.274 min	0.0028 min	0.079 min	
CTNNNAT	500	2.86e-27	1.32e-28	3.66e-28	2.37e-28	2.86e-27
			0.155 min	0.0029 min	0.059 min	
MMMMMMMM	500	1.08e-43	1.07e-39	3.47e-39	1.69e-39	2.71e-38
			0.104 min	0.003 min	0.048 min	
REB1	4000	1.66e-137	9.18e-133	1.19e-131	1.54e-132	1.67e-131
			17.25 min	0.04 min	2.753 min	
CBF1	4000	1.95e-80	9.15e-76	4.62e-75	1.84e-75	1.96e-74
			26.05 min	0.03 min	3.409 min	
UME6	4000	5.42e-88	2.62e-83	3.04e-82	5.11e-83	5.43e-82
			23.81 min	0.03 min	3.374 min	
TYE7	4000	1.62e-43	5.63e-39	2.83e-38	1.39e-38	1.62e-37
			34.25 min	0.02 min	4.05 min	
GCN4	4000	2.04e-50	7.66e-46	4.62e-45	1.80e-45	2.04e-44
			35.43 min	0.03 min	3.95 min	
Puf5	4795	7.91e-85	3.38e-80	5.60e-79	6.95e-80	7.93e-79
			31.51 min	0.027 min	4.51 min	
Pub1	4251	1.49e-84	6.86e-80	1.33e-78	1.37e-79	1.5e-78
			27.74 min	0.033 min	3.81 min	
Pab1	4142	2.46e-11	3.57e-7	5.17e-7	1.37e-6	2.46e-5
			48.46 min	0.007 min	5.41 min	
Khd1	4773	2.74e-20	5.09e-16	1.46e-14	1.73e-15	2.74e-14
			47.58 min	0.015 min	5.84 min	
Nab2	4101	2.09e-11	3.08e-7	1.48e-5	1.18e-6	2.09e-5
			48.7 min	0.016 min	5.34 min	
Vts1	1787	1.44e-10	4.74e-6	1.33e-5	1.4e-5	1.45e-4
			21.94 min	0.003 min	2.07 min	
Pin4	4261	8.16e-14	1.32e-9	8.08e-9	4.83e-9	8.18e-8
			49.38 min	0.011 min	5.48 min	
Nrd1	3947	5.72e-12	9.09e-8	5.71e-6	3.36e-7	5.74e-6
			47.67 min	0.014 min	5.11 min	
Yll032c	2286	1.06e-9	2.62e-5	1.61e-4	8.3e-5	0.001
			35.58 min	0.003 min	2.77 min	

Four bounds are compared over 17 datasets (3 synthetic and 14 biological). For each dataset, the number of sequences (*N*) and the mmHG score are indicated, together with the performance of each bound (in terms of tightness and running time).

### Application in PWM motif search

In this section we discuss mmHG as a framework for assessing the significance of PWM motifs in ranked lists. Given a ranked list of sequences and a PWM motif, by using the mmHG statistics and the bounds introduced earlier, we can assign a *p*-value to represent the significance of that PWM being enriched at the top of the list. To apply this approach for de-novo motif search, one needs to theoretically consider all possible PWMs. However, the search space - when considering position weight matrix motifs – is huge. Assuming the probabilities in the matrix are multiples of 0.1 and the alphabet is of size 4, there are 286^*k*^ possible candidate PWMs of length *k* (since each column must sum to 1, the number of combinations in each column of the matrix is equal to the number of integer solutions for the equation X_1_ + X_2_ + X_3_ + X_4_ = 10, which is $\left(\begin{array}{c} 13\\ 10 \end{array}\right)$). Our approach to navigating in this search space is to narrow the search using the IUPAC alphabet, which considers all possible combinations of letters in the alphabet, and then represent the motif as a PWM based on its actual occurrences in the list. This heuristic approach, called mmHG-Finder, takes as input a ranked list of DNA or RNA sequences and returns significant motifs in PWM format. In cases where sequence ranking is not relevant or not available, it allows the use of positive and negative sets of sequences, searching for enriched motifs in the positive set using the negative set as the background.

We next describe the methodology implemented in mmHG-Finder. The input consists of a ranked list of sequences (or, alternatively, two sets of sequences representing *target* and *background*), as well as the motif width, given as a range [*k*_1_, *k*_2_].

The algorithm:

1. Build a generalized suffix tree for the input sequences.

2. For each *k*=*k*_1_,…,*k*_2_

• Traverse the tree to find all *k*-mers

• Sort the *k*-mers according to their enrichment at the top of the list (this is done using the mHG statistics), as explained in Leibovich *et al.*[8].

• Take the most significant fifty *k*-mers, to be used as starting points for the next step. This set of candidates is chosen such that the members are quite different. Note that this is a heuristic approach and the number 50 is somewhat arbitrary, chosen to succeed in catching the best performing PWMs without heavily paying in complexity.

• For each starting point, we iteratively replace one position in the *k*-mer by considering all possible IUPAC replacements and taking the one that improves the enrichment the most. We repeat this process for all positions several times, and eventually we get a motif in the IUPAC alphabet. We note that given an IUPAC pattern *P*, the occurrences of *P* in the list are extracted efficiently by traversing the paths in the suffix tree that agree with *P*.

• Each IUPAC word is then expanded through a heuristic approach which is based on the Hamming neighbors of that word. Hamming neighbors are added as long as the new addition improves the enrichment *p*-value of the set of words, and as long as the overall similarity between the members in the set does not decrease below a similarity threshold. Since the neighbors are defined as exact words, they usually help in fine-tuning the correct weights of each letter in each position of the resulting PWM. Finally, the expanded motif is converted to a PWM.

3. The PWMs found in the previous step are assessed using the mmHG statistics and the best PWMs are returned as output, together with their *p*-value. The score assigned by a PWM to a string *S* is the maximal score obtained for a substring of *S*. To obtain the likelihood of a substring of length *k* (where *k* is the PWM width), we simply multiply the scores assigned to each letter in each of the positions in that substring.

We provide an efficient implementation of the algorithm described above as publicly available software. Our application takes as input a ranked list of sequences and returns significant PWM motifs. It is compatible with all operating systems and can be freely downloaded from http://bioinfo.cs.technion.ac.il/people/zohar/mmHG-Finder-code/.

**Figure 2:**
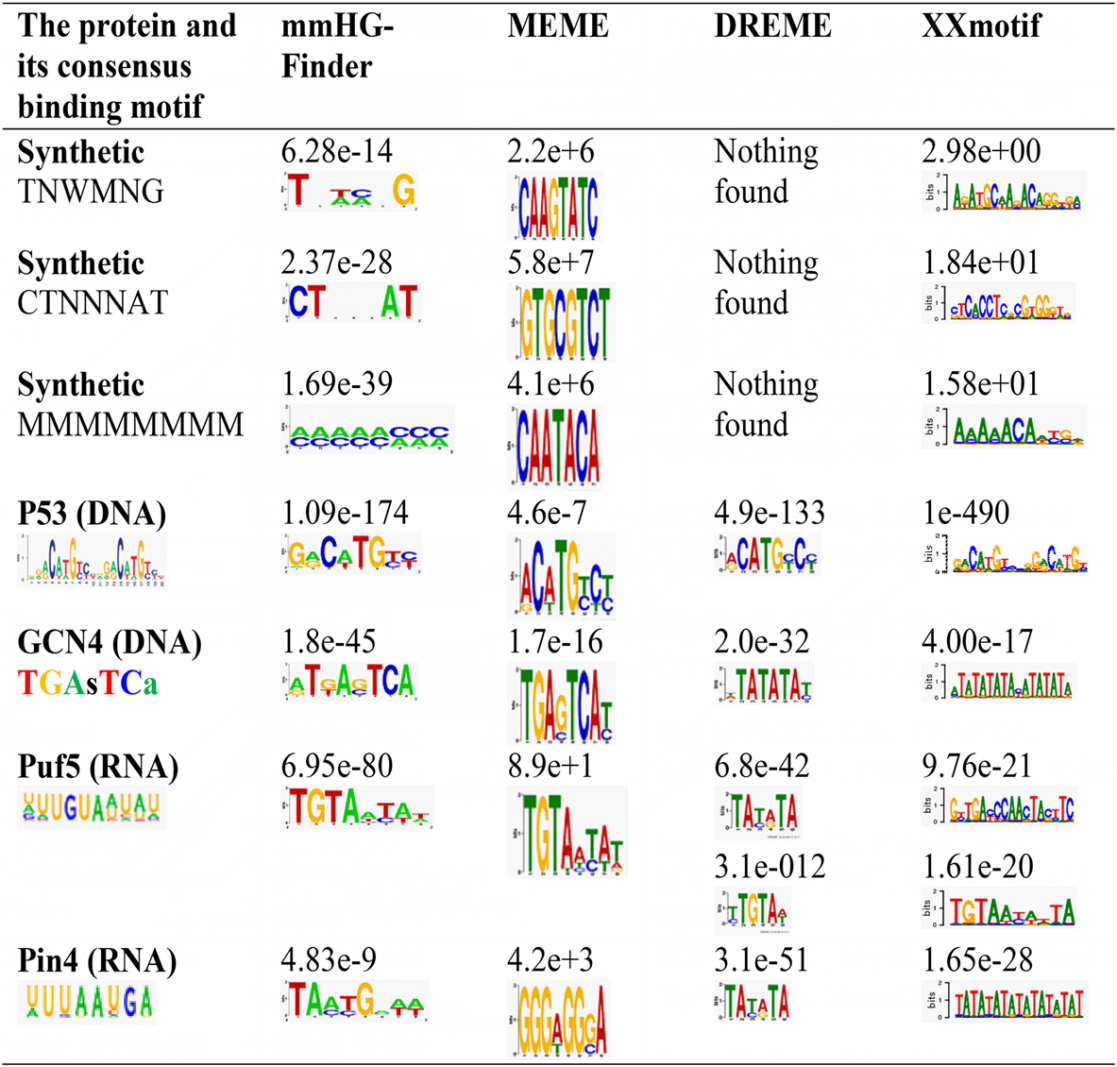
**Comparison between mmHG-Finder and other motif discovery tools.** We evaluated the performance of mmHG-Finder in comparison to other state-of-the-art methods: MEME, DREME and XXmotif. Almost all input examples consisted of ranked lists, except for p53 (comprising target and background sets). Since MEME, DREME, and XXmotif expect to get a target set as input, we converted the ranked lists into target sets by taking the top 100 sequences for MEME (restricted by MEME’s limitation of 60,000 characters) and the top 20% sequences for the other tools. In the synthetic examples the entire ranked lists were taken as they are sufficiently small (to reflect useful comparison with MEME, as the motif is planted in top sequences, we had provided MEME, as input, with the ranking information by adding weights to the sequences, decreasing from 1 to 0 proportionally with the ranking). We used the default parameters in all comparison to other tools (e.g. zero-or-one-occurrence per sequence in MEME) and defined the expected motif length as the range 6 to 8 where possible (specifically, DREME and XXmotif do not have an input parameter for the motif length). Data and consensus motifs for p53 were taken from [31]; for REB1, CBF1, UME6, TYE7, GCN4 from [32]; and for the RNA binding proteins from [33]. Selected results are shown.

To evaluate the performance of mmHG-Finder in comparison to other state-of-the-art methods we ran it on 18 datasets – 3 synthetically generated datasets and 15 generated from high throughput binding experiments (6 transcription factors and 9 RNA-binding proteins). Each synthetic dataset consisted of 500 randomly drawn sequences of length 100. Then, variants of a predefined IUPAC motif were planted at the top 64 sequences of the dataset. We compared the motifs found by mmHG-Finder to those obtained by using three other methods: the standard MEME program [28], DREME [29], and XXmotif [30]. Selected results of this comparison are summarized in Figure 2, and the full output is shown in Additional file 1. Evidently, mmHG-Finder outperformed all the other three tools on the synthetic examples, which contained degenerate motifs. DREME didn’t find the motifs in any case, while MEME and XXmotif found a somewhat similar result in 1 out of the 3 tests. The other 15 examples were taken from DNA and RNA high-throughput experiments [31-33]. For 12 out of these 15 datasets, mmHG-Finder found the motifs which were compatible with the known literature motifs, and as the most significant result. In comparison, DREME found the known consensus in 11 cases; XXmotif detected the literature motif in 9 cases while MEME detected the known motif in 8 cases. In several datasets, such as for Pin4, mmHG-Finder identified the consensus motif while other tools returned repetitive sequences as their top results. The mmHG statistics avoids such spurious results as they typically do not correlate with the measurement driven ranking.

### PWM motif search in long-non-coding RNA sequences

We further analysed a collection of datasets comprising human long-non-coding (lnc) RNAs. LncRNA sequences were extracted and ranked according to the data reported by Cabili et al. [34]. Specifically, a stringent lncRNA set of 4662 loci was tested, where for each locus we know the expression levels in 19 different tissues. From these data we generated 19 lists, each ranked according to tissue-specificity. Given locus *i* and tissue *j*, the tissue specificity score is defined as the difference between the expression of locus *i* in tissue *j* (denoted *exp*_*i*,*j*_) and the mean expression of locus *i* (denoted as *μ*_*i*_). That said difference is measured in terms of the standard deviation of expression in locus *i* (denoted as *σ*_*i*_). Formally:

$$\mathrm{tissue}\;\mathrm{specificity}\;\mathrm{scor}e_{i,j}=\frac{\mathrm{ex}p_{i,j}-\mu_{i}}{\sigma_{i}}\;$$

Calculating the above measure for all tissues reported in [34] yielded 19 ranked lists comprising 4360 lncRNAs (302 loci having standard deviation equal to zero were removed from the analysis). We then conducted three enrichment tests for each of these lists:

1. We searched for de-novo PWM motifs in the promoter sequences of the tissue-specific lncRNAs using mmHG-Finder (introduced in the previous section). Promoter sequences were defined as 1000 bp upstream the transcription start site.

2. We scanned the promoter sequences of the tissue-specific lncRNAs with PWMs corresponding to known transcription factors, downloaded from the JASPAR database [35].

3. Independently of sequence, we calculated the statistical enrichment of measured transcription factor binding events within our lists of loci. Transcription factor binding events within lncRNAs were downloaded from ChIP-Base database [36], which aggregates high-throughput sequencing data taken from hundreds of ChIP-Seq experiments.

**Figure 3:**
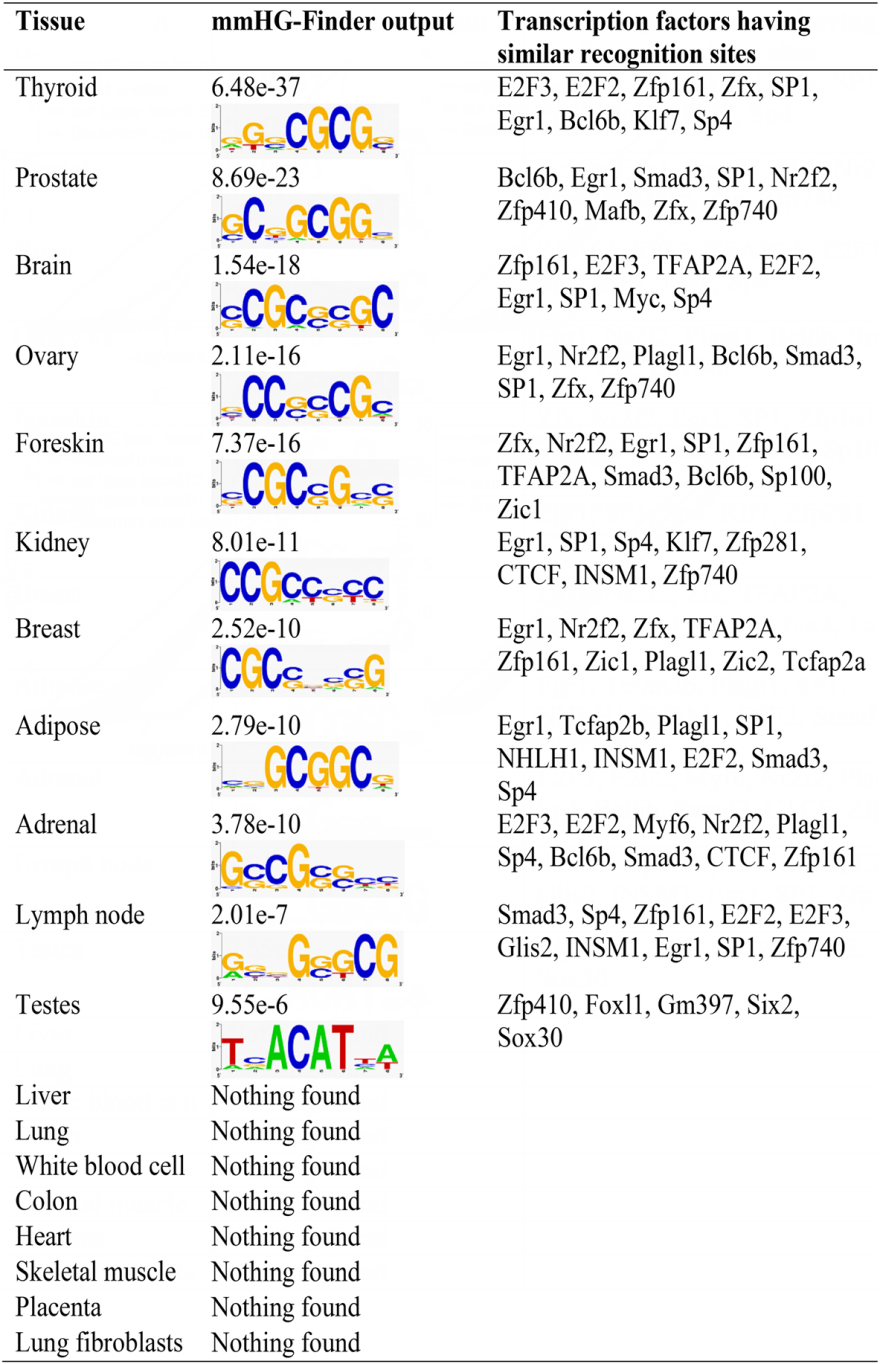
**Motifs in tissue-specific lncRNA promoter sequences.** We analysed the promoter sequences of lncRNAs that are ranked according tissue-specificity. The motifs returned by mmHG-Finder are shown in the figure together with their *p*-value. We compared those motifs to known consensus motifs of transcription factors using TOMTOM [37] (motif database = JASPAR Vertebrates and UniPROBE Mouse) and the most significant results are shown (specifically, all similarity *p*-values are better than 0.018).

Interestingly, almost all the motifs returned by mmHG-Finder were GC-rich (Figure 3). In all three tests, the most significant results were obtained for thyroid-specific and prostate-specific lncRNAs. We further checked whether GC rich sequences are generally enriched amongst the promoter sequences of tissue specific lncRNAs by calculating the mutual enrichment between these two measures. The mutual enrichment between GC content and tissue specificity (Table 2) was the most significant for thyroid (mmHG *p*-value ≤ 3.9∙10^-31^), prostate (5.8∙10^-22^), adrenal (5.5∙10^-20^), brain (1.6∙10^-14^) and ovary (8.8∙10^-12^). Interestingly, Pearson’s correlation between the GC content and the sequence rank was not observed to be strong (strongest correlation coefficient was -0.1), demonstrating that the overall agreement between two measures can be weak even when extremities agree.

**Table 2:** CpG hypo-methylation in tissue-specific lncRNA promoters

**Tissue**	**Mutual enrichment between GC content and tissue-specificity**	**Mutual enrichment between hypo-methylation and tissue-specificity**	
		**Normal/subnormal cells**	**Cancer cells**
Thyroid	3.89e-31	No methylation data	No methylation data
Prostate	5.76e-22	4.16e-11 (PrEC)	0.002 (LNCaP)
Adrenal	5.46e-20	No methylation data	No methylation data
Brain	1.57e-14	1.21e-8 (NH-A)	5.55e-5 (U87)
Ovary	8.80e-12	No methylation data	0.0085 (ovcar-3)
Lymph node	3.64e-6	No methylation data	No methylation data
Adipose	9.25e-6	No methylation data	No methylation data
Foreskin	2.25e-5	0.72 (BJ)	No methylation data
Breast	4.40e-5	5.08e-5 (HMEC)	8.45e-5 (MCF7)
		2.0e-10 (MCF10A)	0.0065 (T-47D)
Kidney	6.34e-5	1.56e-5 (HEK293)	No methylation data
White blood cell	3.78e-4	0.6 (GM12878)	0.21 (Jurkat)
Placenta	0.011	No methylation data	No methylation data
Colon	0.012	No methylation data	1.0 (Caco-2)
Skeletal muscle	0.04	0.34 (SKMC)	No methylation data
Lung	0.33	No methylation data	No methylation data
Heart	1.0	1.0 (HCM)	No methylation data
		1.0 (HCF)	
Liver	1.0	1.0 (Hepatocytes)	1.0 (HepG2)
Testes	1.0	No methylation data	1.0 (NT2-D1)
Lung fibroblasts	1.0	1.0 (IMR90)	No methylation data
		1.0 (AGO4450)	

We calculated the mutual enrichment between DNA hypo-methylation and tissue specificity for the lncRNA promoters. CpG methylation data was taken from UCSC Table Browser [39] (ENCODE/HAIB).

Furthermore, by intersecting the results of the second and the third tests together, we identified transcription factors that may regulate lncRNAs, mainly in thyroid and prostate. This set includes NRF1, E2F1, E2F3, E2F4, E2F6, EGR1, SP1, SP2 and ZBTB33. Moreover, the consensus recognition sites of EGR1, SP1 and E2F3 were found to be similar to the motifs returned by mmHG-Finder in thyroid, prostate and other tissues (Figure 3; the comparison was done using the motif discovery tool TOMTOM [37]). The full output of the second and the third tests are summarized in Additional file 2.

As GC-rich motifs may be associated with CpG methylation, and due to the possible binding of SP1 which has been suggested to protect CpG islands from de novo methylation [17,38], we further tested the association between hypo-methylation and tissue specificity. For that, we downloaded genome wide (450 K) CpG Methylation data from UCSC Table Browser [39] (ENCODE/HAIB). We intersected lncRNA promoter regions with CpG methylation data, and continued only with the 1099 loci that were covered by the methylation experiment. For them, we calculated the mutual enrichment between hypo-methylation and tissue-specificity (the results are summarized in Table 2). Thyroid cells were not covered in this experiment, however two cell lines corresponding to prostate were tested (normal prostate epithelial cell line and cancerous prostate endodermal cell line). We observed that prostate-specific lncRNA promoters were less methylated than non-prostate-specific lncRNAs, and this was much stronger in normal cells than in cancer (mmHG *p*-value ≤ 4.16e-11 in normal prostate cells, and 0.002 in prostate adenocarcinoma cells). We observed strong mutual enrichment between CpG hypo-methylation and tissue-specificity also in brain, ovary, breast and kidney. That is, significant mutual enrichment values were found for tissues where tissue-specific lncRNAs had GC-rich promoter sequences, but these values were not significant for tissues that did not show such GC-bias (heart, liver, testes, and lung fibroblasts). Additionally, in most cases the significance in normal cells was stronger than in cancer, which may be related to changes in methylation patterns acquired during carcinogenesis [40,41].

## Discussion

The assessment of mutual enrichment in ranked lists is often required to support the analysis of biological measurement data, such as in the case of identifying sequence motifs that are involved in regulation processes. Relative ranking can be represented by using permutations over the measured elements. Therefore – the statistical assessment of mutual enrichment can be modelled by characterizing properties of random permutations. Due to the size of the measure space, statistics over S_*N*_, the group of permutations over *N* elements, is difficult to perform and implement. Mutual enrichment is more informative from the point of view of practical biological science than simple correlation measures, as it focuses on the top of the lists and not on the overall agreement, which may be weak even in cases where extremities agree. In this work we derive polynomially computable bounds for the associated tail distribution of mutual enrichment in ranked lists. Namely – we provide methods for computing an upper bound on the *p*-value of mutual enrichment at the top of two permutations uniformly and independently drawn over S_*N*_. Naïve approaches to computing such bounds include variants of the Bonferroni approach. These do not provide tight bounds and may lead to mis-labeling results as non-significant. For several representative datasets, we note that our bound improves the Bonferroni derived *p*-value estimates by a factor of almost 40, on average. Nevertheless, these improvements become relevant only for high *p*-values - for which significant scores should be treated with care anyway. We therefore note that the Bonferroni correction is applicable in many cases, as demonstrated in Table 1. Using our bounds is highly beneficial in borderline cases but is also important in cases where an accurate estimate of the *p*-value is desired, even if nuances do not affect the final biological research conclusions.

We use our statistical/algorithmic framework to support PWM motif searches and demonstrate the application to biological data. We identify motifs that characterize tissue specificity of lncRNA in thyroid and in prostate. Specifically, we find the EGR1 binding motif to be enriched in the promoter regions of lncRNAs which are thyroid-specific. EGR1 was observed to be highly expressed in thyroid (Additional file 1, taken from [36]), consistent with our stronger motif findings. Similarly, EGR1 is highly expressed in adipose tissue and its transcription factor binding sites are enriched in lncRNAs specific to this tissue. We do not have methylation data for the latter two tissues types. However – we do observe the promoters of lncRNAs that are specific to breast to have enriched occurrences of motifs that are similar to EGR1 transcription factor binding sites (*p*-value of similarity according to TOMTOM = 3.52∙10^-5^). EGR1 is also highly expressed in breast. Finally, the promoters of lncRNAs that are specific to breast are less methylated in breast (MCF10A and HMEC cells) than other promoters. This suggests the role of EGR1 in driving tissue differentiation by transcribing tissue-specific lncRNAs and by protecting the associated promoters from methylation. EGR1 has been previously shown to recognize GC-rich consensus sequences located in CpG island promoters of active genes [42]. Generally, we observed that tissue-specific lncRNA promoters tend to be less methylated than those of non-tissue-specific lncRNAs in prostate, brain, ovary, breast and kidney, which may be associated with the GC-rich patterns enriched among their tissue-specific lncRNA promoter sequences.

Threshold-free alternatives to mmHG include the work of McLeay and Bailey, in which a linear regression method is applied [43]. It was shown to achieve high accuracy on a benchmark comprising 237 ChIP-chip datasets, which was higher than all other data driven methods tested, and specifically higher than Spearman’s rank correlation. We note that applying linear regression or Spearman correlation to PWM motif search in ranked lists requires that for significant motifs we observe an overall agreement between the biological measurement and the PWM score. Nevertheless, the standard PWM formulation fails to predict binding affinity when the latter decreases to the point of non-specific binding [44]. In other words, the overall agreement between the PWM score and the binding affinity may be relatively weak. High correlation between the PWM score and the binding affinity needs to hold, in effect, only for sequences demonstrating high-binding affinity with respect to the protein of interest (that is, for sequences that are located at the top of the list) [45]. This weaker relationship is naturally addressed by the mmHG statistics. A combination of mmHG and a linear model, such as suggested in [43], applied to strong binders (top of the list), may yield an even more faithful and informative model.

Future research directions include more extensive application to biological data and the development of tighter and more efficient bounds. Our results show promise in enabling efficient and user-friendly PWM motif search in ranked lists. The software is freely available at http://bioinfo.cs.technion.ac.il/people/zohar/mmHG-Finder-code/. Finally, the full characterization of the distribution of mmHG as a random variable over S_*N*_ remains an open question.

## Conclusions

In this work we developed tight bounds on the tail distribution of mutual enrichment in ranked lists. Our bounds are computable in polynomial time and potentially add to the accuracy of reported results. We demonstrated the utility of mutual enrichment in motif search – specifically, when searching position weight matrix motifs in ranked lists, where the ranking can be according to binding affinity or according to any other biological measurement. Additionally, we used mutual enrichment to study tissue-specific long non-coding RNA regulation, and suggest that tissue-specific lncRNAs are regulated through GC-rich elements located on their promoters, in several tissue types. We hypothesize that these GC-rich patterns are associated with DNA hypo-methylation.

## Competing interests

The authors declare that they have no competing interests.

## Authors’ contributions

LL derived the bounds, developed software and performed analysis. Both authors developed the PWM scoring approach, designed the study and wrote the manuscript. Both authors read and approved the final manuscript.

## Supplementary Material

Additional file 1: Table S1 Comparison between mmHG-Finder and other motif discovery tools. **Figure S1.** EGR1 expression profile. Click here for file

Additional file 2: Table S2 The full output of the second and the third tests (including their intersection) for tissue-specific lncRNAs. Click here for file

## References

[B1] Subramanian A Tamayo P Mootha VK Mukherjee S Ebert BL Gillette MA Paulovich A Pomeroy SL Golub TR Lander ES Mesirov JP Gene set enrichment analysis: a knowledge-based approach for interpreting genome-wide expression profiles Proc Natl Acad Sci U S A 2005 9 15545 15550 10.1073/pnas.0506580102 16199517 PMC1239896

[B2] Eden E Navon R Steinfeld I Lipson D Yakhini Z GOrilla: a tool for discovery and visualization of enriched GO terms in ranked gene lists BMC Bioinformatics 2009 9 48 10.1186/1471-2105-10-48 19192299 PMC2644678

[B3] GOrilla Webserver [http://cbl-gorilla.cs.technion.ac.il/]

[B4] Ragle-Aure M Steinfeld I Baumbusch LO Liestøl K Lipson D Nyberg S Naume B Sahlberg KK Kristensen VN Børresen-Dale A-L Lingjærde OC Yakhini Z Identifying in-trans process associated genes in breast cancer by integrated analysis of copy number and expression data PLoS ONE 2013 9 e53014 10.1371/journal.pone.0053014 23382830 PMC3559658

[B5] Akavia UD Litvin O Kim J Sanchez-Garcia F Kotliar D Causton HC Pochanard P Mozes E Garraway LA Pe’er D An integrated approach to uncover drivers of cancer Cell 2010 9 1005 1017 10.1016/j.cell.2010.11.013 21129771 PMC3013278

[B6] Dehan E Ben-Dor A Liao W Lipson D Frimer H Rienstein S Simansky D Krupsky M Yaron P Friedman E Rechavi G Perlman M Aviram-Goldring A Izraeli S Bittner M Yakhini Z Kaminski N Chromosomal aberrations and gene expression profiles in non-small cell lung cancer Lung Cancer 2007 9 175 184 10.1016/j.lungcan.2006.12.010 17258348

[B7] Al-Shahrour F Díaz-Uriarte R Dopazo J FatiGO: a web tool for finding significant associations of gene ontology terms with groups of genes Bioinformatics 2004 9 578 580 10.1093/bioinformatics/btg455 14990455

[B8] Leibovich L Yakhini Z Efficient motif search in ranked lists and applications to variable gap motifs Nucleic Acids Res 2012 9 5832 5847 10.1093/nar/gks206 22416066 PMC3401424

[B9] Leibovich L Paz I Yakhini Z Mandel-Gutfreund Y DRIMust: a web server for discovering rank imbalanced motifs using suffix trees Nucleic Acids Res 2013 9 W174 W179 10.1093/nar/gkt407 23685432 PMC3692051

[B10] DRIMust Webserver [http://drimust.technion.ac.il/]

[B11] Steinfeld I Navon R Ach R Yakhini Z miRNA target enrichment analysis reveals directly active miRNAs in health and disease Nucleic Acids Res 2013 9 e45 e45 10.1093/nar/gks1142 23209027 PMC3561970

[B12] miTEA Webserver [http://cbl-gorilla.cs.technion.ac.il/miTEA/]

[B13] Enerly E Steinfeld I Kleivi K Leivonen S-K Ragle-Aure M Russnes HG Rønneberg JA Johnsen H Navon R Rødland E Mäkelä R Naume B Perälä M Kallioniemi O Kristensen VN Yakhini Z Børresen-Dale A-L miRNA-mRNA integrated analysis reveals roles for miRNAs in primary breast tumors PLoS ONE 2011 9 e16915 10.1371/journal.pone.0016915 21364938 PMC3043070

[B14] Plis SM Weisend MP Damaraju E Eichele T Mayer A Clark VP Lane T Calhoun VD Effective connectivity analysis of fMRI and MEG data collected under identical paradigms Comput Biol Med 2011 9 1156 1165 10.1016/j.compbiomed.2011.04.011 21592468 PMC3174276

[B15] Eden E Lipson D Yogev S Yakhini Z Discovering motifs in ranked lists of DNA sequences PLoS Comput Biol 2007 9 e39 10.1371/journal.pcbi.0030039 17381235 PMC1829477

[B16] Steinfeld I Navon R Ardigò D Zavaroni I Yakhini Z Clinically driven semi-supervised class discovery in gene expression data Bioinformatics 2008 9 i90 i97 10.1093/bioinformatics/btn279 18689846

[B17] Straussman R Nejman D Roberts D Steinfeld I Blum B Benvenisty N Simon I Yakhini Z Cedar H Developmental programming of CpG island methylation profiles in the human genome Nat Struct Mol Biol 2009 9 564 571 10.1038/nsmb.1594 19377480

[B18] Lee B-K Bhinge AA Iyer VR Wide-ranging functions of E2F4 in transcriptional activation and repression revealed by genome-wide analysis Nucleic Acids Res 2011 9 3558 3573 10.1093/nar/gkq1313 21247883 PMC3089461

[B19] Rhee Ho S Pugh BF Comprehensive genome-wide protein-DNA interactions detected at single-nucleotide resolution Cell 2011 9 1408 1419 10.1016/j.cell.2011.11.013 22153082 PMC3243364

[B20] Lebedeva S Jens M Theil K Schwanhäusser B Selbach M Landthaler M Rajewsky N Transcriptome-wide analysis of regulatory interactions of the RNA-binding protein HuR Molecular Cell 2011 9 340 352 10.1016/j.molcel.2011.06.008 21723171

[B21] Hafner M Landthaler M Burger L Khorshid M Hausser J Berninger P Rothballer A Ascano M Jr Jungkamp A-C Munschauer M Ulrich A Wardle GS Dewell S Zavolan M Tuschl T Transcriptome-wide identification of RNA-binding protein and MicroRNA target sites by PAR-CLIP Cell 2010 9 129 141 10.1016/j.cell.2010.03.009 20371350 PMC2861495

[B22] Staden R Computer methods to locate signals in nucleic acid sequences Nucleic Acids Res 1984 9 505 519 10.1093/nar/12.1Part2.505 6364039 PMC321067

[B23] Stormo GD Schneider TD Gold L Quantitative analysis of the relationship between nucleotide sequence and functional activity Nucleic Acids Res 1986 9 6661 6679 10.1093/nar/14.16.6661 3092188 PMC311672

[B24] Hertz GZ Stormo GD Identifying DNA and protein patterns with statistically significant alignments of multiple sequences Bioinformatics 1999 9 563 577 10.1093/bioinformatics/15.7.563 10487864

[B25] Tompa M Li N Bailey TL Church GM De Moor B Eskin E Favorov AV Frith MC Fu Y Kent WJ Makeev VJ Mironov AA Noble WS Pavesi G Pesole G Regnier M Simonis N Sinha S Thijs G van Helden J Vandenbogaert M Weng Z Workman C Ye C Zhu Z Assessing computational tools for the discovery of transcription factor binding sites Nat Biotech 2005 9 137 144 10.1038/nbt1053 15637633

[B26] Sinha S On counting position weight matrix matches in a sequence, with application to discriminative motif finding Bioinformatics 2006 9 e454 e463 10.1093/bioinformatics/btl227 16873507

[B27] Abramowitz M Stegun IA Handbook of Mathematical Functions with Formulas, Graphs, and Mathematical Tables 1964 New York: Dover Publications, Inc.

[B28] Bailey TL Elkan C Unsupervised learning of multiple motifs in biopolymers using expectation maximization Mach Learn 1995 9 51 80

[B29] Bailey TL DREME: motif discovery in transcription factor ChIP-seq data Bioinformatics 2011 9 1653 1659 10.1093/bioinformatics/btr261 21543442 PMC3106199

[B30] Luehr S Hartmann H Söding J The XXmotif web server for eXhaustive, weight matriX-based motif discovery in nucleotide sequences Nucleic Acids Res 2012 9 W104 W109 10.1093/nar/gks602 22693218 PMC3394272

[B31] Smeenk L van Heeringen SJ Koeppel M van Driel MA Bartels SJJ Akkers RC Denissov S Stunnenberg HG Lohrum M Characterization of genome-wide p53-binding sites upon stress response Nucleic Acids Res 2008 9 3639 3654 10.1093/nar/gkn232 18474530 PMC2441782

[B32] Harbison CT Gordon DB Lee TI Rinaldi NJ Macisaac KD Danford TW Hannett NM Tagne J-B Reynolds DB Yoo J Jennings EG Zeitlinger J Pokholok DK Kellis M Rolfe PA Takusagawa KT Lander ES Gifford DK Fraenkel E Young RA Transcriptional regulatory code of a eukaryotic genome Nature 2004 9 99 104 10.1038/nature02800 15343339 PMC3006441

[B33] Hogan DJ Riordan DP Gerber AP Herschlag D Brown PO Diverse RNA-binding proteins interact with functionally related sets of RNAs. Suggesting an extensive regulatory system PLoS Biol 2008 9 e255 10.1371/journal.pbio.0060255 18959479 PMC2573929

[B34] Cabili MN Trapnell C Goff L Koziol M Tazon-Vega B Regev A Rinn JL Integrative annotation of human large intergenic noncoding RNAs reveals global properties and specific subclasses Genes Dev 2011 9 1915 1927 10.1101/gad.17446611 21890647 PMC3185964

[B35] Mathelier A Zhao X Zhang AW Parcy F Worsley-Hunt R Arenillas DJ Buchman S Chen C-y Chou A Ienasescu H Lim J Shyr C Tan G Zhou M Lenhard B Sandelin A Wasserman WW JASPAR 2014: an extensively expanded and updated open-access database of transcription factor binding profiles Nucleic Acids Res 2013 9 D142 D147 24194598 10.1093/nar/gkt997 PMC3965086

[B36] Yang J-H Li J-H Jiang S Zhou H Qu L-H ChIPBase: a database for decoding the transcriptional regulation of long non-coding RNA and microRNA genes from ChIP-Seq data Nucleic Acids Res 2013 9 D177 D187 10.1093/nar/gks1060 23161675 PMC3531181

[B37] Gupta S Stamatoyannopoulos J Bailey T Noble W Quantifying similarity between motifs Genome Biol 2007 9 R24 10.1186/gb-2007-8-2-r24 17324271 PMC1852410

[B38] Brandeis M Frank D Keshet I Siegfried Z Mendelsohn M Names A Temper V Razin A Cedar H Sp1 elements protect a CpG island from de novo methylation Nature 1994 9 435 438 10.1038/371435a0 8090226

[B39] UCSC Table Browser [http://genome.ucsc.edu/cgi-bin/hgTables?command=start]

[B40] Bert SA Robinson MD Strbenac D Statham AL Song JZ Hulf T Sutherland RL Coolen MW Stirzaker C Clark SJ Regional activation of the cancer genome by long-range epigenetic remodeling Cancer Cell 2013 9 9 22 10.1016/j.ccr.2012.11.006 23245995

[B41] Nejman D Straussman R Steinfeld I Ruvolo M Roberts D Yakhini Z Cedar H Molecular rules governing de novo methylation in cancer Cancer Res 2014 9 1475 1483 10.1158/0008-5472.CAN-13-3042 24453003

[B42] Kubosaki A Tomaru Y Tagami M Arner E Miura H Suzuki T Suzuki M Suzuki H Hayashizaki Y Genome-wide investigation of in vivo EGR-1 binding sites in monocytic differentiation Genome Biol 2009 9 R41 10.1186/gb-2009-10-4-r41 19374776 PMC2688932

[B43] McLeay R Bailey T Motif enrichment analysis: a unified framework and an evaluation on ChIP data BMC Bioinformatics 2010 9 165 10.1186/1471-2105-11-165 20356413 PMC2868005

[B44] Frank DE Saecker RM Bond JP Capp MW Tsodikov OV Melcher SE Levandoski MM Record MT Thermodynamics of the interactions of lac repressor with variants of the symmetric lac operator: effects of converting a consensus site to a non-specific site J Mol Biol 1997 9 1186 1206 10.1006/jmbi.1997.0920 9150406

[B45] Benos PV Lapedes AS Stormo GD Is there a code for protein-DNA recognition? Probab(ilistical)ly Bioessays 2002 9 466 475 10.1002/bies.10073 12001270

