# Supplementary material for: Mutual enrichment in ranked lists and the statistical assessment of position weight matrix motifs
Source: Algorithms Mol Biol. 2014 Apr 5;9:11. doi: 10.1186/1748-7188-9-11 (PMC4021615; doi:10.1186/1748-7188-9-11)
Supplement: Additional file 1: Table S1 — Comparison between mmHG-Finder and other motif discovery tools. Figure S1. EGR1 expression profile. [file 1748-7188-9-11-S1.pdf]

Supplementary Table 1 – Comparison between mmHG-Finder and other motif discovery tools

We evaluated the performance of mmHG-Finder in comparison to other state-of-the-art methods: MEME, DREME and XXmotif over 18 datasets. This table is more comprehensive than Table 1 and contains it.

The protein and its consensus binding motif	mmHG-Finder results	MEME results	DREME results	XXmotif results
**Synthetic** TNWMNG	6.28e-14 *	2.2e+6	Nothing found	2.98e+00
**Synthetic** CTNNNAT	2.37e-28 *	5.8e+7	Nothing found	1.84e+01
**Synthetic** MMMMMMMM	1.69e-39 *	4.1e+6 *	Nothing found	1.58e+01 *
**P53 (DNA)**	1.09e-174 *	4.6e-7 *	4.9e-133 *	1e-490 *
**REB1 (DNA)** **TTACCCG**	1.54e-132 *	4.3e-88 *	1.1e-80 *	3.15e-89 *
**CBF1 (DNA)** **tCACGTG**	1.84e-75 *	2.3e-59 *	1.1e-39<br>2.0e-023 (4th best) *	5.00e-15<br>2.72e-10 (3rd best) *
**UME6 (DNA)** **taGCCGCCsa**	5.11e-83 *	3.8e-76 *	3.1e-45<br>5.1e-030 (3rd best) *	3.52e-47 *
**TYE7 (DNA)** **tCACGTGa**	1.39e-38 *	2.1e-30 *	7.3e-43<br>1.7e-018 (4th best) *	9.26e-21<br>1.11e-10 (3rd best) *
**GCN4 (DNA)** **TGAsTCa**	1.8e-45 *	1.7e-16 *	2.0e-32<br>2.5e-005 (9th best) *	4.00e-17
**Puf5 (RNA)**	6.95e-80 *	8.9e+1 *	6.8e-42<br>3.1e-012 *	9.76e-21<br>1.61e-20 *

**Pub1 (RNA)** 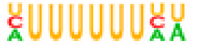	1.37e-79 * 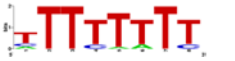	1.2e+4 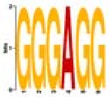	2.1e-36 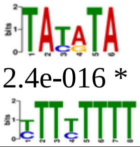 2.4e-016 *	5.73e-18 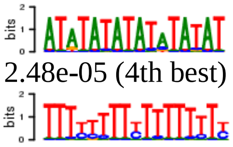 2.48e-05 (4th best) *
**Pab1 (RNA)** 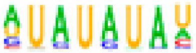	1.37e-6 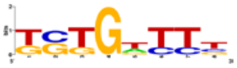	4.9e+3 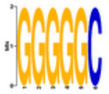	5.4e-45 * 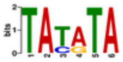	5.89e-28 * 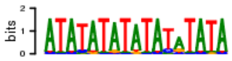
**Khd1 (RNA)** 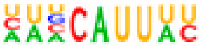	1.73e-15 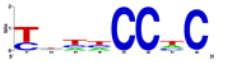	1.8e+3 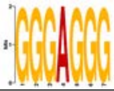	1.2e-45 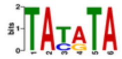	1.24e-26 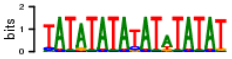
**Nab2 (RNA)** 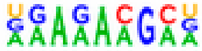	1.18e-6 * 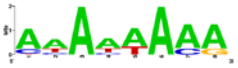	3.8e+3 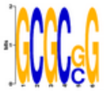	1.0e-39 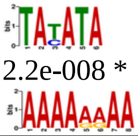 2.2e-008 *	8.16e-29 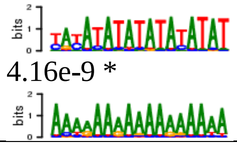 4.16e-9 *
**Vts1 (RNA)** 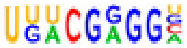	1.4e-5 * 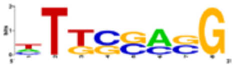	1.2e+4 * 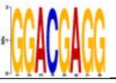	1.0e-14 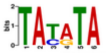	1.08e-8 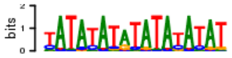
**Pin4 (RNA)** 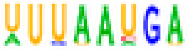	4.83e-9 * 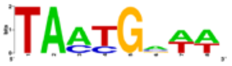	4.2e+3 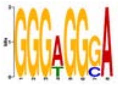	3.1e-51 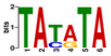	1.65e-28 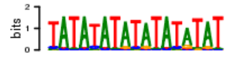
**Nrd1 (RNA)** 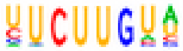	3.36e-7 * 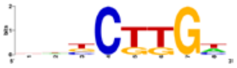	3.4e+3 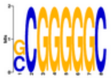	4.1e-44 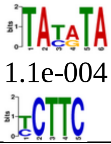 1.1e-004 (3rd best) *	4.15e-20 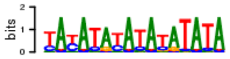
**YLL032C (RNA)** 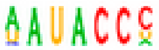	8.3e-5 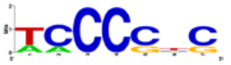	7.3e+3 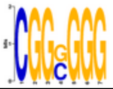	3.4e-24 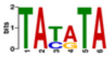	9.54e-14 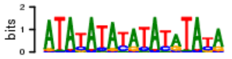

* indicates success in identifying the consensus motif

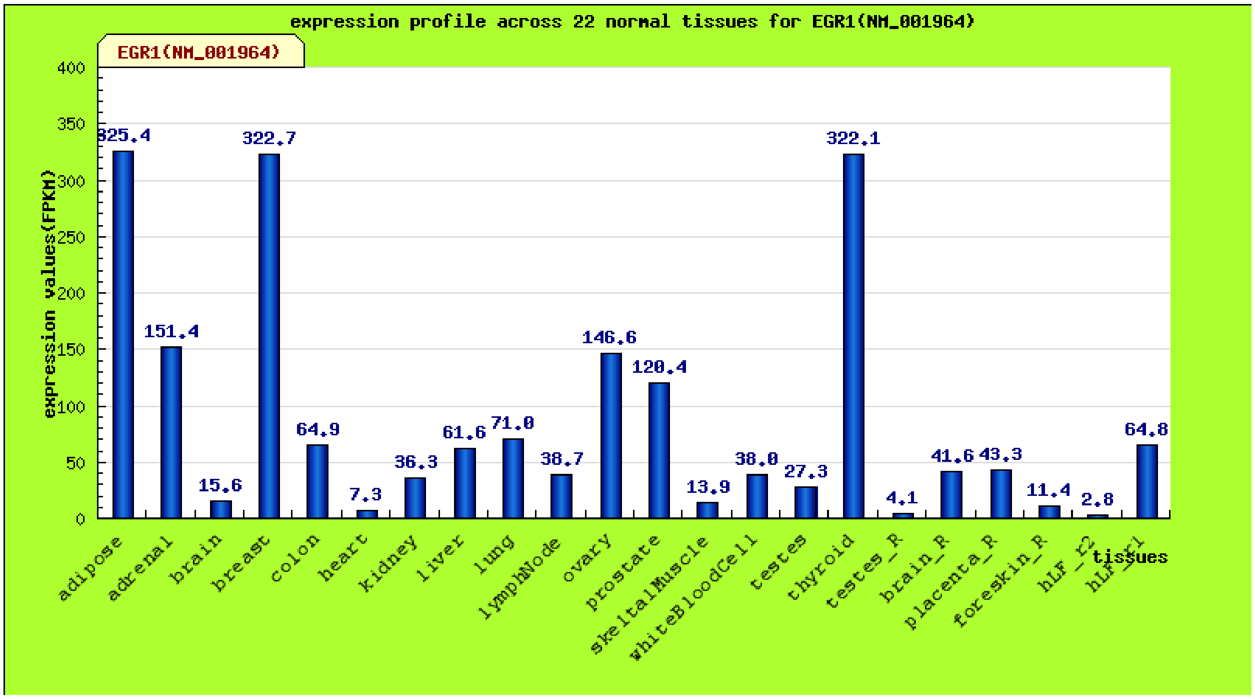

Supplementary Figure 1 – EGR1 expression profile

The expression profile of EGR1 in 22 tissues is shown below. As can be seen, EGR1 is highly expressed in thyroid, adipose and breast. We note that the expression values for the lncRNAs were obtained for the same set of tissues (in our analysis we excluded replicates and focused on 19 distinct tissues).
